# On the Duration of Life in the Foetus in Utero, after the Mother’s Death

**Published:** 1879-09

**Authors:** 


### ON THE DURATION OF LIFE IN THE FŒTUS IN UTERO AFTER THE MOTHER'S DEATH.

THIS question has been carefully investigated by C. Garezky, in his inaugural dissertation, St. Petersburgh, 1878. He has collected 379 cases, in which the Cæsarean operation was performed after death; 308 infants were extracted dead, 37 showed signs of life, 34 were born alive; but of these only five remained alive for some time. The author then gives a sketch of Breslau's experiments on animals, and sums up his conclusions as follows: 1. The fœtus undoubtedly survives the sudden death of the mother. 2. If it can be extracted in the course of the first six minutes, it may be born alive. 3. Six to ten minutes after the mother's death, the child may still be alive, though slightly asphyxiated. 4. Ten to twenty minutes after death, the infant is highly asphyxiated. 5. In a great many cases the infants are either highly asphyxiated or dead after the first minute. 6. The shorter the time is which elapses between the cause of the mother's death, and the ceasing of the cardiac action, the longer the fœtus remains alive. 7. If the mother's death has been caused by some quickly acting poison, the chances for the child's life are greater than when it has been brought on by other causes.—*British Medical Journal, June, 1879.*

